# Butorphanol or a Combination of Ketamine and Xylazine Do Not Interfere With Arrhythmogenic Parameters in Agoutis (*Dasyprocta prymnolopha*) Obtained Through High‐Resolution Electrocardiogram

**DOI:** 10.1002/jez.2922

**Published:** 2025-04-09

**Authors:** Charlys Rhands Coelho de Moura, Hermínio José da Rocha Neto, Regina Lucia dos Santos Silva, Nayla Maria da Silva Rezende Amorim, José Lindenberg Rocha Sarmento, Miguel Ferreira Cavalcante Filho, Wanderson Gabriel Gomes de Melo, Dayseanny de Oliveira Bezerra, Maria Acelina Martins Carvalho, Napoleão Martins Argolo Neto

**Affiliations:** ^1^ Federal University of Piauí, Ministro Petrônio Portela University Campus (UFPI) Teresina Brazil; ^2^ Advanced Campus of José de Freitas, Federal Institute of Education, Science and Technology of Piauí José de Freitas Brazil

**Keywords:** cardiology, physiology, wild animals

## Abstract

Introduction/Objectives: Agoutis is a medium‐sized wild rodent with potential for use as an experimental model. This study aimed to evaluate physiological parameters of arrhythmogenesis in this species, by HR‐ECG and VCG techniques, under physical and pharmacological containment (ketamine‐xylazine and butorphanol). Materials and methods: Eight agouti in which the physiological parameters of arrhythmogenesis were evaluated by HR‐ECG and VCG techniques, under physical and pharmacological containment. We evaluated cardiac and pulmonary foci sounds, made femoral pulse inspection, body score analysis, gestational evaluation, echocardiographic examination, blood pressure measurement, and conventional electrocardiography. Results: The non‐sedated agoutis exhibited QRS duration of 93.25 ± 9.51, LAS < 40 μV of 28.50 ± 4.65 and RMS of 93.50 ± 29.75. The value of QRS duration decreased in animals treated with ketamine‐xylazine and increased in those receiving butorphanol. The non‐sedated agoutis exhibited QRS duration of 93.25 ± 9.51, LAS < 40 μV of 28.50 ± 4.65 and RMS of 93.50 ± 29.75. The value of QRS duration decreased in animals treated with ketamine‐xylazine and increased in those receiving butorphanol. The sedation protocols did not cause alterations for LAS < 40 μV and RMS. The sQRST value in non‐sedated animals was 54.20° ± 43.63 and the administration of ketamine‐xylazine increased this index to 102.87° ± 53.57, while butorphanol did not induce alteration. The mean systolic, diastolic, and mean arterial pressures differed between physical and pharmacological restraints. Both pharmacological constraints reduced the blood pressure of the agoutis, analogously and mildly. Conclusion: The adoption of sedation protocols using the ketamine‐xylazine association and butorphanol did not interfere with HR‐ECG values and exhibited minimal VCG data and blood pressure changes.

## Summary

- This is the first study evaluating arrhythmogenesis parameters in sedated agoutis.
- Evaluation of sedated agoutis using high‐resolution ECG and vectorcardiography.
- Sedation with ketamine‐xylazine or butorphanol did not affect arrhythmogenesis parameters in agoutis.
- Results suggest that ketamine‐xylazine and butorphanol are safe for agouti sedation.

**Abbreviations**
DBP: Diastolic blood pressure
HR‐ECG: High‐resolution electrocardiogram
LAS < of 40 Μv: High frequency and low amplitude signal duration in the final portion of the filtered QRS complex
MAP: Mean arterial pressure
QRS duration: Total QRS complex duration
RMS: Square root of the mean voltage of the last 40 ms of the filtered QRS complex
SBP: Mean systolic blood pressure
VCG: Vectocardiography

## 1 Introduction

The development and improvement of experimental models in cardiology is a constant and current demand, given the requirements of reliable mimicry of the human species’ cardiac physiopathology conditions. Moreover, its main objective is to suppress the ethical barrier of not intervening primarily in *anima nobile* (Ferreira et al. 2005). However, the different morphophysiological parameters between inferior animals and humans and between animals of other species, breeds and sex, limit the embracing applicability of cardiological models in both human and veterinary medicine (Scheffer et al. 2013).

In cardiological models, contemporary studies indicate the use of complex physiological variables to predict arrhythmogenesis (Oehler et al. 2014; Chamas 2011; Turrini et al. 1999) and are referred advanced non‐invasive techniques such as high‐resolution electrocardiogram and vectocardiography to obtain them as the vanguard of clinical cardiology in the early detection of diseases in the human species (Man et al. 2011; Narayanaswamy 2002).

High‐resolution electrocardiogram (HR‐ECG) is used to assess the risk of ventricular arrhythmias and sudden death resulting from several heart diseases, such as channelopathies and arrhythmogenic right ventricular dysplasia. This technique demonstrates a positive correlation between post‐late potentials and the occurrence of sustained ventricular tachycardia (Turrini et al. 1999; Folino et al. 2006; Nava 2000).

In veterinary medicine, HR‐ECG has been used in a restricted manner in clinical situations, such as in canine patients with dilated cardiomyopathy, for example (Calvert 1998). A recent study reported high sensitivity and specificity of HR‐ECG to predict the probability of sudden death of cardiac origin in Boxer canine patients (Spier and Meurs 2004). Due to a lack of data in animals, we identified a need for studies that specify normality and morbidity indices for different animal species.

Vectocardiography (VCG) was widely studied in the 1970s in the human species, and its applicability was limited by the technology available in the following two decades (Maheshwari et al. 2016). It has currently regained scientific notoriety due to its relevance to the precise diagnosis of the Brundle branch and fascicle lesions of the specialized cardiac conduction system (Pastore et al. 2016). This technique allows early diagnosis of supraventricular arrhythmias’ origin and the identification of intraventricular electrical conductivity alterations in humans (Kück et al. 2018). However, there are still no reports of the use of this technique in domestic and wild animals.

The restriction to the use of advanced diagnostic techniques in animals, in the long term, may compromise the advancement of human cardiology, resulting from the need to use animal models to understand the physiopathogenic, electromechanical, and hemodynamic mechanisms involved in heart diseases. The benefit gained from animal models’ use is mutual to humans and animals that may share new therapeutic guidelines, providing higher expectations and quality of life for both (Suzuki et al. 2008).

Ideally, the animal model should have morphophysiological characteristics that allow the adoption of methodologies and equipment for investigation. The pathological phenomenon and the morbid condition induced should be similar to the morbidity of natural occurrence in the species in which data extrapolation is sought (Ferreira et al. 2005).

Thus, all models will present limitations dependent on the object of study. Therefore, the canine model has ethical barriers regarding its use due to its acceptance in today's society as a family member. In turn, despite the tremendous physiological similarity with humans, pigs have a high cost of maintenance in confinement. Murines are isogenic among individuals, favoring the safety of the reproducibility of scientific data, but require sophisticated equipment, adapted to its low body size, in which the acquisition cost is high (Fagundes and Taha 2004).

Among wild rodents, agoutis (*Dasyprocta prymnolopha*) have a medium size, classified in the suborder Histricomorpha, family Dasyproctidae and found from Mexico to South America (Cubas et al. 2014). Many studies have been conducted on the species, evaluating its morphology, nutritional and reproductive aspects, as a model for nephrology studies and donor of stem cells (Cabral et al. 2012; Carreiro et al. 2018; Júnior et al. 2012; Wise et al. 2014). However, there are few data on this species’ cardiovascular system, limiting the clinical and surgical approach in captive‐bred animals. One of the main limitations of using HR‐ECG and VCG in animals is the mandatory pharmacological restraint. Veterinary medical routine can use several as mild sedatives, especially the association between ketamine hydrochloride and xylazine hydrochloride or opioid use, due to its extensive use (Spinosa and Spinosa 1991).

Veterinarians have commonly adopted the ketamine hydrochloride (Dopalen, Vetbrands, São Paulo, São Paulo, 12327‐673, Brazil) and xylazine hydrochloride (Anasedan, Vetbrands, São Paulo, São Paulo, 12327‐673, Brazil) association due to suppressing the profound depressant effect of xylazine, caused by ketamine and ketamine‐induced catatonia suppression, by the action of xylazine. We more easily achieved a safe sedative effect when applied associated than alone (Andrade et al. 2002).

Butorphanol tartrate (Torbugesic, Fort Dodge, Campinas, São Paulo, 13604‐798 Brazil) is a synthetic opioid of sedative action that presents lower respiratory depression and dysphoria than other μ‐opioid agonists (Wagner et al. 2002) and, induces medullary analgesia and sedation between 2 and 4 h after 15 min of parenteral administration (Massone 2011).

Thus, this study aimed to evaluate the physiological parameters of arrhythmogenesis in adult agoutis (*Dasyprocta prymnolopha*) by HR‐ECG and VCG techniques, under physical restraint and comparative pharmacological restraint, using the association ketamine‐xylazine or butorphanol, by parenteral route.

## 2 Materials and Methods

### 2.1 Animals

This study was authorized by the Ethics Committee on Animal Experimentation of the Federal University of Piauí (UFPI) under n.381/17, following the guidelines of the Brazilian College of Animal Experimentation and the Authorization and Information System in Biodiversity (SISBIO) of the Chico Mendes Institute for Biodiversity Conservation (ICMBio) n°. 69544‐1.

Eight agoutis (*Dasyprocta prymnolopha*), four males and four females were used, with an average weight of 2.5 ± 1.5 kg, with a mean age of 6 ± 1.2 years, raised in captivity, in the Center for studies, production and preservation of wild animals (NEPPAS) of UFPI, under registration with the Brazilian Institute of the Environment and Renewable Natural Resources (IBAMA) n°. 02/08‐618. The subcutaneous electronic chip helped the identification of all animals kept in community bays of 12m^2^, fed with commercial feed (Minimum crude protein ‐ 18%, Etherium extract ‐ 1.5%, Fibrous matter ‐ 12%, Calcium ‐ 1.3% and Phosphorus ‐ 0.4%), in addition to corn, fruits, tubers, regional vegetables, and water ad libitum. The number of animals and sample distribution between males and females was based on the current NEPPAS/UFPI squad availability.

### 2.2 Clinical Screening Analysis

Over a year and a half, the animals were previously conditioned to the management with a single handler, favoring that it performed physical restraint with minimal effort. Each animal was submitted to physical restraint, only once, for clinical screening tests.

We evaluated cardiac and pulmonary foci sounds, made femoral pulse inspection, body score analysis, gestational evaluation, echocardiographic examination, blood pressure measurement, and conventional electrocardiography. We performed cardiac auscultation, and femoral pulse evaluation conventionally and evaluated the body score using the Body Condition Score System.

To verify possible pregnant agoutis, we performed Ultrasound analysis as described above 45. For echocardiography, the MYLaB30 Gold equipment (Esaote™, Viale Cristoforo Colombo, 49, 20090 Trezzano sul Naviglio MI, Itália) with a 7.5–11 MHz scan range transducer was used under the right and left lateral decubitus position.

A conventional electrocardiographic examination was simultaneously performed for 5 min, in the right lateral decubitus position, using a digital electrocardiograph (DMS®, Tatuapé, São Paulo, São Paulo, 03067‐010, Brazil) with CardioScan software Resting version 4.00.

Only clinically healthy animals were selected, with no alterations in complementary tests and nonpregnant.

### 2.3 Experimental Management

The agoutis were identified and managed separately, in the morning, fasting, submitted to three protocols, and 30 days between them. For the first protocol, each animal was subjected to physical restraint, only once, in the right lateral decubitus position. After 30 days, each animal was submitted to mild sedation, administering 15 mg/kg of ketamine hydrochloride and 2 mg/kg of xylazine hydrochloride applied together, intramuscularly, posterior region of the femoral quadriceps. Similarly, each animal was submitted to the third protocol after the same period, administering 0.3 mg/kg of butorphanol tarthate, using the same application pathway. Data collection began 30 min after applying the drugs, and the animals were followed up until the complete recovery of sedation.

The butorphanol tartrate dose was obtained by allometric extrapolation, using the mean specific metabolic rate of agoutis (0.0504 Kcal/g/day), the specific metabolic rate of dogs with 10 Kg (393.63 Kcal/g/day) and the dose of the drug for canines (0.3 mg/kg) (Brito et al. 2010).

### 2.4 High‐Resolution Electrocardiography (HR‐ECG)

The animals were positioned in the right lateral decubitus position, the electrodes placed in the region between the fifth and sixth right and left intercostal space of the xiphoid cartilage and manubrium and in the areas of the dorsal spinous process of the seventh thoracic vertebra and cardiac apex, in the topography of precordial shock location (Frank X, Y, Z orthogonal leads). Six minutes of tracing were acquired, using two passband filters of 25–250 Hz and 40–250 Hz to reduce noise to values below 1 μV and 0.7 μV, respectively, for each type of filter (DMS Brasil® CardioScan Resting 4.00 software).

The variables “total QRS complex duration (QRS duration),” “high frequency and low amplitude signal duration in the final portion of the filtered QRS complex (LAS < of 40 μV)” and the “square root of the mean voltage of the last 40 ms of the filtered QRS complex (RMS)” were measured. Means of each variable were obtained for each animal, in both sexes, under physical and pharmacological containment (ketamine‐xylazine and butorphanol).

### 2.5 Vectocardiography (VCG)

Simultaneously the acquisition of the HR‐ECG, under even decubitus and even positioning of electrodes (orthogonal leads X, Y, Z of Frank), vectocardiography data were obtained. The vectors were evaluated in the frontal, sagittal and horizontal planes, considering the three‐dimensional spatial distribution corresponding to each level in the right, left, upper, lower, front and back directions.

The angles of QRS (aQRS), T, P and QRS‐T, the frontal planar QRS‐T index (fpQRST), the calculated spatial QRS‐T (sQRST) index and the orientation of the inscription of the QRS complex, P and T wave in the frontal, right and horizontal planes were measured. Bayley's hexaxial system (Bayley 1943) evaluated the ventricular depolarization axis variation in the frontal plane and the frontal, horizontal and sagittal planes by the Frank's method (Frank 1956).

### 2.6 Noninvasive Blood Pressure (NIBP)

To measure blood pressure (BP), we used PetMap® oscillometry equipment (Ramsey Medical Inc. USA). Cuffs were chosen according to the limbs (width of 40% of the diameter), using as standard, the right anterior limb, positioning the animals in the left lateral decubitus position. We performed eight sequential measurements to determine the mean systolic and diastolic pressures. The mean per animal was obtained, for both sexes, under physical and pharmacological restraint (ketamine‐xylazine and butorphanol).

### 2.7 Statistical Analysis

For each analysis variable, the means of the animals and the general mean were obtained, with their respective standard deviations and coefficients of variation. We compared the data collected for the three containment protocols, which are considered treatments (physical containment, sedation with hydrochloride combination of ketamine‐xylazine and sedation with butorphanol tarthate).

We choose a completely randomized experimental design composed of two groups (males vs. females), three treatments (types of containment) with four replications each, using the Student's T‐test with the significance of 5%. As there are no reference data for the agoutis’ species, we also considered the “physical restraint” treatment as control, using the comparison of the other groups.

We performed the analyses with the bioestat software® version 5.3 (free software, Mamirauá Institute, Brazil).

## 3 Results

In the literature survey, we did not identify specific studies regarding the techniques of HR‐ECG, VCG and NIBP, only one report with the associated use of ketamine hydrochloride ‐ xylazine hydrochloride was found, and no work evidenced with the use of butorphanol tartrate in the agoutis’ species in the last 10 years (2009–2019).

No animal presented general clinical and cardiovascular system alterations in the semiological and complimentary screening tests. No relevant changes were identified in conventional electrocardiographic and echocardiographic examinations. Body score was defined with a mean value of 4.0 ± 1.8 kg, considering both sexes.

The HR‐ECG evaluation identified the mean values of, LAS < of 40 μV and RMS in non‐sedated healthy agoutis, with alert and calm consciousness level, was 93.25 ± 9.51, 28.50 ± 4.65, and 93.50 ± 29.75, respectively. We did not observe significant differences between males and females.

Among the treatments, only the variable QRS duration differed significantly (*p* ≤ 0.05), identified as the highest average in animals treated with butorphanol, followed by the treatments “physical containment” and “ketamine‐xylazine,” respectively. The difference between the highest mean for QRS duration (butorphanol) and physical restraint (control) was 0.25 ms, while the mean difference between the ketamine‐xylazine and butorphanol groups was 24.75 ms (Table 1).

**TABLE 1:** High‐resolution electrocardiography (HR‐ECG).

	PHYSICAL RESTRAINT	KETAMINE‐XYLAZINE	BUTORPHANOL
QRS *(ms)*	46.36 ± 7.04^a^	40.50 ± 7.57^a^	47.00 ± 6.76^a^
QRS duration *(ms)*	93.25 ± 9.51^a^	68.75 ± 8.20^b^	93,50 ± 8.66^c^
LAS *(ms)*	28.50 ± 4.65^a^	21.75 ± 3.80^a^	23.62 ± 3.11^a^
RMS *(µV)*	93.50 ± 29.75^a^	91.12 ± 35.20^a^	127.38 ± 21.11^a^

*Note*: Average values of the total QRS complex duration (QRS duration), duration of the high frequency and low amplitude signal in the final portion of the filtered QRS complex (LAS < 40 µV) and the square root of the average voltage of the last 40 ms of the complex Filtered QRS (RMS) of agouti (*Dasyprocta prymnolopha*) (*n* = 8) raised in captivity, submitted to physical and pharmacological restraint. Equal letters do not differ at 5% significance (*Student's T test*).

Abbreviation: ms = millisecond.

**FIGURE 1:**
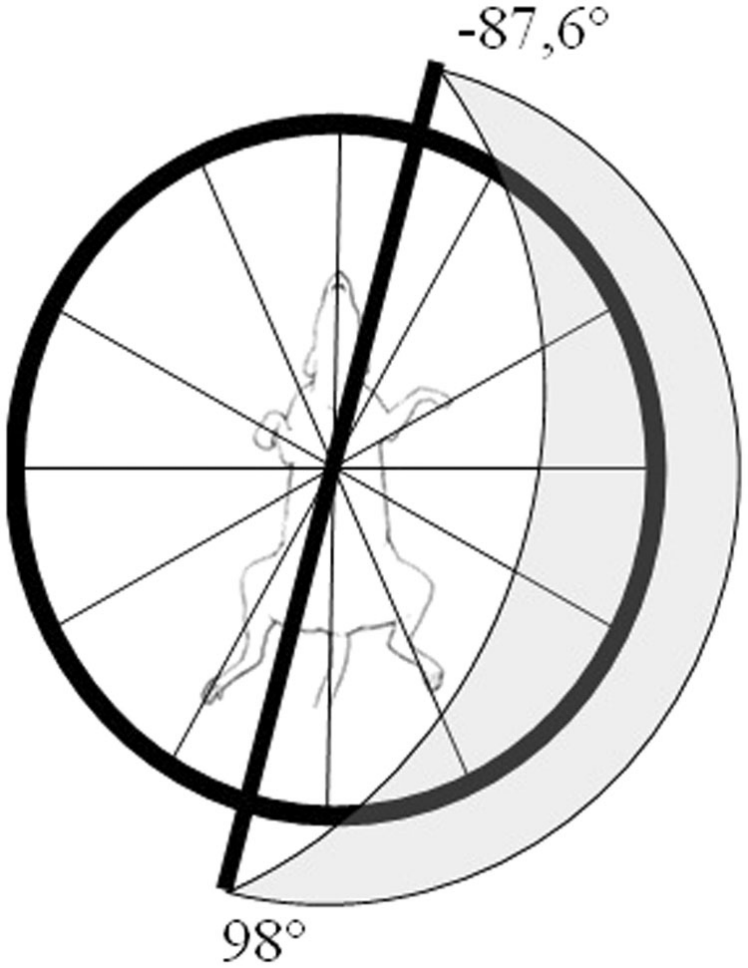
Graphical representation of the mean QRS angle vector (aQRS) in the frontal plane by Bayley's hexaxial system (1943). The amplitude of the ventricular depolarization vector of agoutis (*Dasyprocta prymnolopha*), under physical restraint, revealed orientation to the left, up and down, ranging from −0087.6° to 98° in all animals (DMS Brasil® software CardioScan Resting 4.00).

**FIGURE 2:**
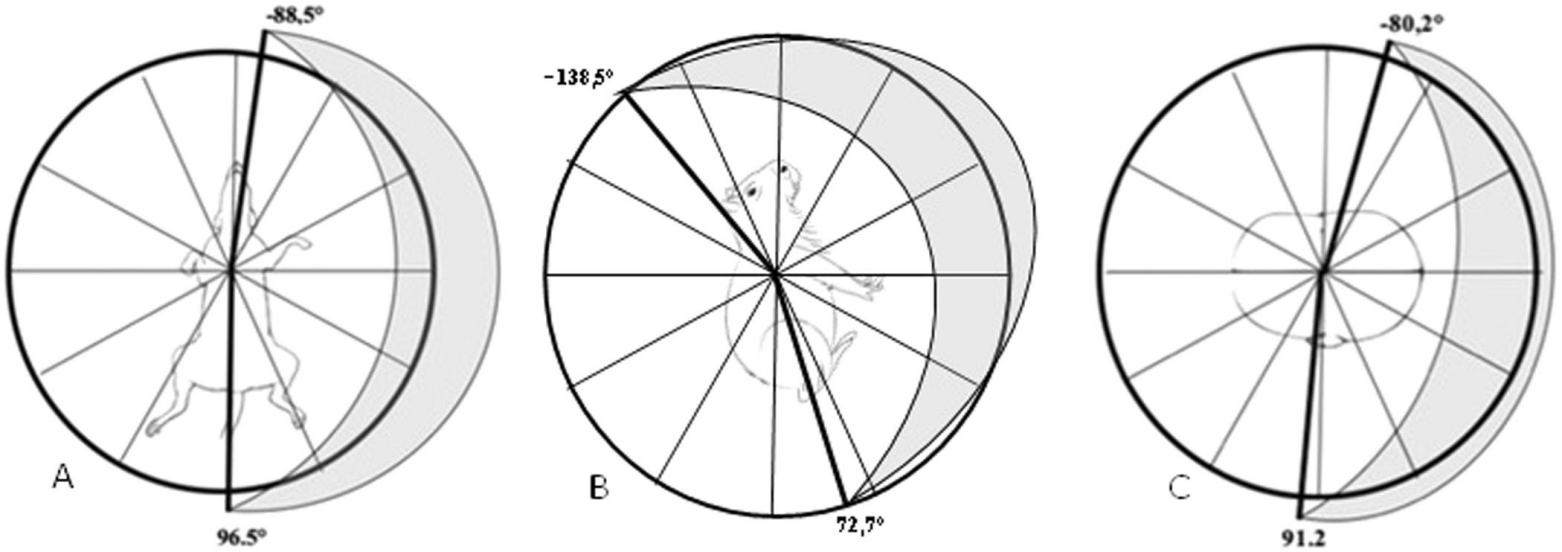
Graphic representation of the mean QRS angle vector (*a*QRS) of agoutis (*Dasyprocta prymnolopha*), under physical restraint, by Frank's method (1956). (A) Frontal plane. The vector amplitude of ventricular depolarization revealed orientation to the left, up and down, ranging from −88.5° to 96.5°. (B) Sagittal plane. Predominantly forward, up and down, ranging from 72.7° to −138.5°. (C) Horizontal plane. Predominantly to the left, back, and forward, ranging from −80.2° to 91.2° (DMS Brasil® software CardioScan Resting 4.00).

The measurement of the mean aQRS vector, by Bailey's hexaxial system, identified the lowest value of −87.6° and 98° for all agoutis, in both sexes under physical restraint (Figure 1). The application of Frank's traditional method identified a predominance of vector direction in the frontal plane down and to the left; in the sagittal plane forward and downward; and in the horizontal plane, backward and to the left (Table 2) (Figure 2), with no significant variation (*p* ≥ 0.05) for both sexes, under physical and pharmacological restraint (ketamine‐xylazine and butorphanol). The mean values of the vector inscription of the T, P, and QRS‐T angles in the frontal, sagittal, and horizontal planes are shown in Table 2.

**TABLE 2:** Mean values of QRS angles (*a*QRS), T, P, and QRS‐T, in the frontal, sagittal and horizontal planes of agoutis (*Dasyprocta prymnolopha*) (*n* = 8), raised in captivity, submitted to physical restraint.

		PHYSICAL RESTRAINT
FRONTAL PLANE	QRS ANGLE	4.00 ± 92.48
	P ANGLE	85.75 ± 72.17
	T ANGLE	77.87 ± 90.86
	QRS‐T ANGLE	79.25 ± 67.04
SAGITTAL PLANE	QRS ANGLE	−32.88 ± 105.57
	P ANGLE	17.75 ± 120.97
	T ANGLE	77.50 ± 69.49
	QRS‐T ANGLE	37.37 ± 45.08
HORIZONTAL PLANE	QRS ANGLE	5.50 ± 85.7
	P ANGLE	6.50 ± 91.24
	T ANGLE	24.12 ± 110.93
	QRS‐T ANGLE	46.00 ± 54.80

**FIGURE 3:**
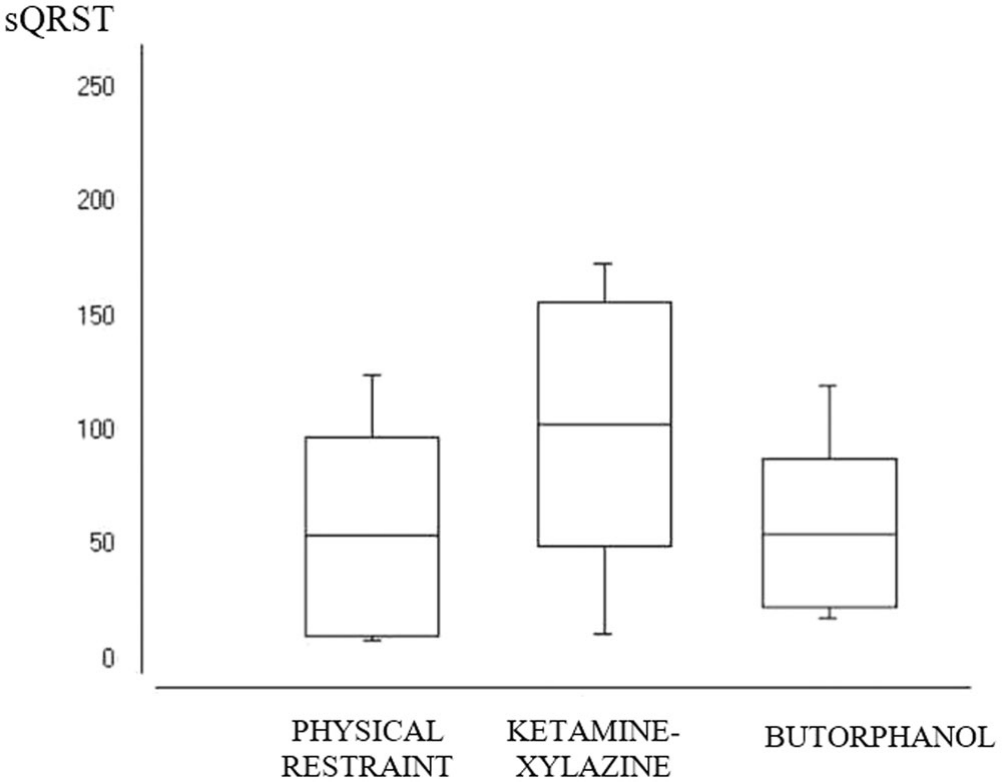
Distribution of the calculated spatial index of QRS‐T (sQRST), in agoutis (*Dasyprocta prymnolopha*) (*n* = 8), raised in captivity, submitted to physical and pharmacology restraint at 5% significance (Software Bioestat® 5.0).

The evaluated agoutis’ sQRST value, with an alert and calm level of consciousness, under physical restraint, was 54.20° ± 43.63. Pharmacological containment with ketamine‐xylazine increased this index to 102.87° ± 53.57 (*p* ≤ 0.05), while butorphanol did not induce alteration (*p* ≥ 0.05). However, compared to the ketamine‐xylazine protocol, butorphanol reduced the sQRST to 55.750° ± 32.59 (*p* ≤ 0.05) (Figure 3).

The mean systolic blood pressure (SBP), diastolic blood pressure (DBP), mean arterial pressure (MAP) and heart rate (HR) of the agoutis differed significantly (*p* ≤ 0.05) between physical restraint and pharmacological restraints (ketamine‐xylazine and butorphanol). No differences (*p* ≥ 0.05) of MAP were identified between ketamine‐xylazine and butorphanol restraints (Table 3).

**Table 3:** The mean systolic blood pressure (SBP), diastolic blood pressure (DBP), mean arterial pressure (MAP) and heart rate (HR) of agouti (*Dasyprocta prymnolopha*) (*n* = 8) raised in captivity, subjected to physical and pharmacological containment.

	PHYSICAL RESTRAINT	KETAMINE‐XYLAZINE	BUTORPHANOL
SBP *(mmHg)*	147.50 ± 8.45^a^	128.75 ± 7.90^b^	131.25 ± 8.34^b^
DBP *(mmHg)*	95.62 ± 6.78^a^	84.37 ± 8.21^b^	84.47 ± 8.12^b^
MAP *(mmHg)*	112.88 ± 6.33^a^	99.12 ± 7.82^b^	100.00 ± 6.92^b^
HR *(bpm)*	162.88 ± 11.83^a^	140.00 ± 8.01^b^	127.38 ± 21.11^b^

*Note:* Equal letters do not differ at 5% significance (*Student's T test*).

## 4 Discussion

This study is the first of the physiological parameters of arrhythmogenesis by HR‐ECG and VCG in the agoutis’ species (*Dasyprocta prymnolopha*) in veterinary medicine. The use of early diagnosis techniques for ARCGAr and VCG arrhythmias is still recent (Coelho et al. 2013). The prospection of the last 10 years demonstrated the lack of studies of physiological patterns with the agoutis’ species. This fact limits the possibility of comparative analysis of the data obtained but demonstrates their relevance for this species, which constitutes a frontier area of knowledge, using an unconventional animal model.

All agoutis used in this experiment recovered completely from sedation and were reintroduced to the squad. Both sedation protocols using the combination ketamine‐xylazine and butorphanol proved to be safe, without deleterious changes in vital parameters and subsequent rapid recovery. The safety of the ketamine‐xylazine association protocol had been previously demonstrated in agoutis (Diniz et al. 2017). However, there were, to date, no data regarding the use of butorphanol in this species. It is essential to reiterate both protocols’ safety since captive‐bred wild animals may present secondary metabolic alterations resulting from drug administration. However, we did not observe such complications in this study.

The acquisition of HR‐ECG data in agoutis, regardless of sex (*p* ≥ 0.05), identified QRS duration values (68.7 ms) similar to those described in a previous study for the same species under the same restraint conditions (50 ± 7.1 ms) (Diniz et al. 2013), as well as QRS duration, LAS < 40 μV, and RMS (Table 1) values comparable to those measured in the human species. The physiological values for humans recommended by the American College of Cardiology are QRS duration lower than 120 ms, LAS < 40 μV less than 40 ms, and RMS higher than 25 μV 6. In dogs, these values vary according to the breed and size of the animal. In boxer, HR‐ECG values are reported with a mean QRS duration of 82.16 ms, LAS < 40 μV of 24.84 ms, and RMS 473.56 μV 40. In Dobermann Pinscher, these variables are described in less than 75 ms, less than 26 ms and greater than 117 μV, respectively (Calvert et al. 1997). Thus, the shared values of QRS duration, LAS < 40 μV, and RMS for the canine species are entirely distinct from the human species. This fact denotes the relevance of the data obtained in agoutis, given their approximation to human's reference values. Moreover, the absence of data published in the scientific literature for HR‐ECG variables in mice and rats also contributes to the understanding that the agoutis species may be considered a viable rodent model for studies on the subject.

The QRS duration variable is related to the propagation velocity of the intraventricular electrical impulse and, subsequently, the speed of fast sodium channels opening and (Jaye et al. 2010). Therefore, the higher the phase 0 ascent rate of cardiomyocyte depolarization and the higher amplitude of the action potential, the more efficiently these cells transmit the cardiac impulse to the next cell, and the faster the electrical conduction along the cardiac fiber (Sperelakis and Ramasamy 2010). Thus, it is suggestive that the values identified in the non‐sedated agoutis indicate that the passage of the action potential by the muscular fibers of the ventricle occurs as quickly as described in humans. As the evaluation of the dynamics of cardiac sodium channels was not the object of this study, further investigations of this variable in agoutis are required to validate this hypothesis.

Both sedation protocols altered the ventricular conduction velocity (*p* ≤ 0.05) concerning the alert animals, under physical restraint (Table 1). The observed effect of the ketamine‐xylazine association on QRS duration may be related to the intrinsic adrenergic characteristic of ketamine. According to these authors, ketamine induces circulating catecholamines and, consequently, the stimulation of the receptor pathways α1 and ß1, increasing cardiac work. Such observation allows us the syllogism that the increase in cardiac work contributed to the rise in the velocity of ventricular conduction, given these variables physiological interdependence. Diametrically, the effect of butorphanol on QRS duration is probably due to the stimulation of κ‐ opioid receptors, reducing sympathetic tone and heart rate (Watkins‐Pitchford 2010). We believed that this effect might have led to increased ventricular conduction velocity in animals sedated with butorphanol.

Despite these variations, no severe rhythmic changes were observed, such as tachyarrhythmia, bradyarrhythmia, or ventricular ectopy. The maintenance of cardiac rhythmicity suggests that both protocols are safe for *agoutis* sedation. Besides, none of the sedation protocols raised the QRS duration values to close to 120 ms, in which, based on human parameters, it would favor the presence of arrhythmogenic substrates as late potentials. As the results demonstrated that non‐sedated agoutis have higher mean values of QRS duration than dogs, this analogy does not compare these species.

Las values < 40 μV in agoutis indicate probable integrity of specialized cardiac conduction tissue since values above 38 ms are commonly related to the delay in the conduction of the electrical impulse in likely areas of ventricular scar, inducing re‐entry mechanisms that favor the dyssynchrony of electrical formation and conduction during the refractory period of depolarization, called late potentials, in humans (Breithardt et al. 1991). Contemporary studies had incriminated the presence of these potentials as a predisposing factor to the production of ventricular arrhythmias and sudden death (Benchimol Barbosa et al. 2002).

The similarity between the variable LAS < 40 μV obtained in alert agoutis, under physical containment, with the human species’ values, arouses possible electrophysiological analogy between these species. Thus, in dogs, the LAS interval < 40 µV is stricter than in humans (Chamas et al. 2016), canine models for studies of late potentials could induce false‐positive results since the tolerance interval for delayed ventricular cardiac impulse in humans is more significant than in dogs. This fact, at least hypothetically, could be minimized in models using agoutis.

The observation of the RMS of the agoutis indicates a low probability of occurrences of late potentials, corroborating the statements of LAS < 40 μV, since this variable is inversely proportional to the presence of late potentials (Benchimol Barbosa et al. 2002). As this is not a physiological variable but a statistical variable, it is dependent on the variation of LAS < 40 μV and, therefore, its analysis is evaluation subordinated.

The pharmacological containment protocols did not induce significant changes (*p* ≥ 0.05) for the variables LAS < 40 μV and RMS, both compared to each other and physical containment. This observation reiterates the safety of drugs for this species under the conditions of this study.

The cardiac vector's direction identified in the agoutis was similar to that described in the scientific literature for mammals (Hasan et al. 2012). This condition reflects the obviousness of the agoutis’ cardiac topography, in which the cardiac apex is pointed to the left, the sinus node, located at the base of the right atrium and the left ventricle has a higher number of cardiac fibers (Singh et al. 1996). Thus, the depolarization vector path follows the septum depolarization sequence (ECG Q wave) and later to the ventricular walls, inducing the vector data described in this study.

The pfQRST obtained in agoutis differed from the values described in humans (MacFarlane 2012), representing an idiosyncrasy of the species. According to these authors, humans have, on average, pfQRST of 12°. The Q‐T interval obtained by conventional ECG can explain the upper index obtained in this study (Table 2). As the pfQRST presents electrophysiological correlation with the Q‐T gap and the agoutis show a longer duration of this interval (Diniz et al. 2017) compared to humans (Mady et al. 1985; De Moura et al. 2013; Zacché et al. 2017), physiologically, the values obtained seem to us to be consistent.

The pfQRST has been widely applied in predicting mortality due to severe arrhythmias in humans (Oehler et al. 2014; Filho and Chaves 2000), without evidence of equivalent animal research to date. We believed that the pfQRST obtained in the agoutis of this study is the first in veterinary cardiology.

The agoutis’ pfQRST and the sQRST, was within the normality standards recommended for the human species (Kück et al. 2018). According to these authors, humans have mean sQRST below 105°, just like this study (Figure 3). There are no standard data so far in veterinary scientific literature. A single contemporary study conducted in rats identified that the progressive increase in sQRST, in the interval between 49° ± 46 to 146° ± 45, was pathologically associated with cardiac remodeling (Henkens et al. 2007). This information corroborates the human species’ patterns, for which angles greater than 135° (Kück et al. 2018) are considered pathological.

The increase in sQRST pharmacologically induced by the ketamine‐xylazine association (Figure 3) probably results from the increase in myocardial repolarization amplitude (T), caused by the adrenergic rise in myocardial oxygen consumption, mediated by ketamine (Zanos et al. 2018). As sQRST is derived from the amplitude of depolarization (QRS) and repolarization (T), this index seems to reflect a cardiac electrophysiological adaptation to the drug under the conditions of this study. Thus, the pharmacological increase of sQRST in agoutis did not exceed the limit established in the human species, nor did it induce tachyarrhythmias under this study's conditions. Subsequently, cell oxidation studies in cardiac electrophysiology may contribute to clarify these hypotheses.

The administration of butorphanol did not interfere with sQRST concerning non‐sedated animals, despite the potential cholinergic effect commonly reported in mammals, with subsequent reduction of sympathetic tone (Watkins‐Pitchford 2010). This observation suggests that the possible pharmacological reduction of myocardial repolarization amplitude (T) at a dose of 0.03 mg/kg did not reach magnitude to induce sQRST decrease in this study's animals. This effect, hypothetically, could contribute to minimizing the possibility of occurrences of ventricular tachyarrhythmias. We expect that this study's results favor the establishment of new research that investigates the action of butorphanol in animal models of induced ventricular tachyarrhythmias.

The evaluation of aQRS and T angle indicated values in the agoutis utterly distinct from the human species. The human species is the only one to date that presents reference values established for aQRS of 33° ± 17, 39° ± 30, and 327° ± 13 in the frontal, sagittal and horizontal planes, respectively (Ishikawa et al. 1967). The discrepancy observed between aQRS and T angle between agoutis and humans is irrelevant, because these angles do not have a direct predictive application to the study of arrhythmogenesis in humans, commonly used to obtain pfQRST and sQRST.

The values found in this study's agoutis, with an alert level of consciousness, under physical restraint, were similar to the values referenced for the canine and feline species. These species present SBP below 140 mmHg, DBP below 95 mmHg, and MAP below 110 mmHg (Brown et al. 2007), equivalent to that observed in agoutis (Table 3). Regarding humans, the values obtained in agoutis were hugely discrepant, presenting higher for SBP, DBP, and MAP. This observation raises possible limitations of the agoutis’ species for studies involving blood pressure as a model for the human species, although it presents the potential for an alternative model to dogs and cats.

However, the most relevant aspect of the study of blood pressure in agoutis is its correlation for valid VCG data validation. Blood pressure can behave as an intervening variable influencing vetocardiographic variables, since hypertensive conditions can induce left ventricular remodeling, with subsequent change in cardiac vectors’ angle. As the animals in this study were previously submitted to screening tests to rule out this condition's presence, it is believed that the values obtained are physiological to the species.

Both pharmacological restraints reduced the agoutis’ blood pressure, similarly (*p* ≥ 0.05) (Table 3). Although studies refer to the ketamine‐xylazine protocol as transient hypertensive potential (Bertozzo et al. 2008), we observed the opposite effect in this study's conditions. It is believed that perhaps the parasympathomimetic effects of xylazine have outweighed the adrenergic action of ketamine in agoutis, as described earlier in domestic animals (Spinosa and Spinosa 1991). Contrary, studies commonly refer to butorphanol as an antagonistic effect of sympathetic tone (Watkins‐Pitchford 2010). and expect to identify blood pressure reduction. However, the reduction in blood pressure observed was mild in both sedation protocols, denoting its use's safety under the conditions of this study.

## 5 Conclusion

Agoutis represents a potential animal model for arrhythmogenesis studies, as it presents HR‐ECG and sQRST data, under the healthy condition and with an alert and calm level of consciousness, similar to the human species. It also exhibits blood pressure variation under the same conditions, similar to dogs and cats. The adoption of sedation protocols using the ketamine‐xylazine association and butorphanol did not interfere with HR‐ECG values and exhibited minimal VCG data and blood pressure changes.

## Author Contributions

**Charlys Rhands Coelho de Moura:** study concept or design, data collection, data analysis or interpretation, writing the paper, final approval of the version to be published, drafting the work or revising it critically for important intellectual content. **Regina Lucia dos Santos Silva, Wanderson Gabriel Gomes de Melo, Miguel Ferreira Cavalcante Filho,** and **Nayla Maria da Silva Rezende Amorim:** writing the paper, investigation, drafting the work or revising it critically for important intellectual content, final approval of the version to be published. **Hermínio José da Rocha Neto** and **José Lindenberg Rocha Sarmento:** writing the paper, drafting the work or revising it critically for important intellectual content, final approval of the version to be published. **Maria Acelina Martins Carvalho, Napoleão Martins Argolo Neto,** and **Dayseanny de Oliveira Bezerra:** study concept or design, data collection, data analysis or interpretation, writing the paper, final approval of the version to be published, drafting the work or revising it critically for important intellectual content.

## Ethics Statement

This study was approved by the Animal Ethics Committee (CEUA/UFPI) under protocol number 381/17. All experiments were conducted in accordance with the ethical standards and guidelines established by the committee.

## Conflicts of Interest

The authors declare no conflicts of interest.

## Data Availability

The data that support the findings of this study are available from the corresponding author upon reasonable request.

## References

[jez2922-bib-0001] Andrade, A. , S. Pinto , and R. Oliveira . 2002. Criação e manejo de cobaias [Relatório técnico].

[jez2922-bib-0002] Bayley, R. H. 1943. “On Certain Applications of Modern Electrocardiographic Theory to the Interpretation of Electrocardiograms Which Indicate Myocardial Disease.” American Heart Journal 26: 769–831. 10.1016/S0002-8703(43)90288-1.

[jez2922-bib-0003] Benchimol Barbosa, P. R. , M. O. Sousa , E. C. Barbosa , A. S. Bomfim , P. Ginefra , and J. Nadal . 2002. “Analysis of the Prevalence of Ventricular Late Potentials in the Late Phase of Myocardial Infarction Based on the Site of Infarction.” Arquivos Brasileiros de Cardiologia 78: 352–363. 10.1590/S0066-782X2002000400002. 12011951

[jez2922-bib-0004] Bertozzo, D. , R. E. Freitas , F. Reis , R. Reis , D. S. Santos , and W. A. Souza . 2008. “Contenção química em animais silvestres.” Revista Científica Eletrônica de Medicina Veterinária 11: 1–6.

[jez2922-bib-0005] Breithardt, G. , M. E. Cain , N. el‐Sherif , et al. 1991. “Standards for Analysis of Ventricular Late Potentials Using High‐Resolution or Signal‐Averaged Electrocardiography. A Statement by a Task Force Committee of the European Society of Cardiology, the American Heart Association, and the American College of Cardiology.” Circulation 83: 1481–1488. 2013173 10.1161/01.cir.83.4.1481

[jez2922-bib-0006] Brito, H. F. V. , R. R. Lange , J. R. Pachaly , and I. Deconto . 2010. “Determinação da taxa metabólica basal em cutias, *Dasyprocta azarae*, por calorimetria indireta.” Pesquisa Veterinária Brasileira 30: 471–478. 10.1590/S0100-736X2010000600001.

[jez2922-bib-0007] Brown, S. , C. Atkins , R. Bagley , et al. 2007. “Guidelines for the Identification, Evaluation, and Management of Systemic Hypertension in Dogs and Cats.” Journal of Veterinary Internal Medicine 21: 542–558. 17552466 10.1892/0891-6640(2007)21[542:gftiea]2.0.co;2

[jez2922-bib-0008] Cabral, R. M. , M. S. Ferraz , M. S. Rizzo , et al. 2012. “Kidney Injury and Cell Therapy: Preclinical Study.” Microscopy Research and Technique 75: 566–570. 10.1002/jemt.21092. 22619746

[jez2922-bib-0009] Calvert, C. A. 1998. “High‐Resolution Electrocardiography.” Veterinary Clinics of North America: Small Animal Practice 28: 1429–1447. 10.1016/S0195-5616(98)50130-1. 10098246

[jez2922-bib-0010] Calvert, C. A. , G. Hall , G. Jacobs , and C. Pickus . 1997. “Clinical and Pathologic Findings in Doberman Pinschers With Occult Cardiomyopathy That Died Suddenly or Developed Congestive Heart Failure: 54 Cases (1984–1991).” Journal of the American Veterinary Medical Association 210: 505–511. 10.2460/javma.1997.210.04.505. 9040836

[jez2922-bib-0011] Carreiro, A. N. , J. A. R. A. Diniz , J. G. Souza , et al. 2018. “Ovary and Vaginal Epithelium Dynamics During the Estrous Cycle in *Dasyprocta prymnolopha* Wagler, 1831: Ultrasound and Cytological Examinations.” Journal of Veterinary Science 19: 446–451. 10.4142/jvs.2018.19.3.446. 29284211 PMC5974526

[jez2922-bib-0012] Chamas, P. P. C. 2011. Estudo do eletrocardiograma ambulatorial, eletrocardiograma de alta resolução HR‐ECG e variabilidade da frequência cardíaca como indicadores prognósticos na cardiomiopatia arritmogênica de cães Boxer [Tese de doutorado, Universidade de São Paulo]. Universidade de São Paulo.

[jez2922-bib-0013] Chamas, P. P. C. , V. M. C. Oliveira , F. L. Yamaki , G. T. Goldfeder , and M. H. M. A. Larsson . 2016. “Valor prognóstico da variabilidade da frequência cardíaca e da eletrocardiografia ambulatorial em cães Boxer com cardiomiopatia arritmogênica do ventrículo direito.” Arquivo Brasileiro de Medicina Veterinária e Zootecnia 68: 1219–1227. 10.1590/1678-4162-8383.

[jez2922-bib-0014] Coelho, M. , R. Muzzi , A. Silva , G. Oberlender , and B. Henrique . 2013. “Strain e strain rate bidimensionais – novas perspectivas na cardiologia veterinária.” Revista Científica Eletrônica de Medicina Veterinária 11: 14.

[jez2922-bib-0015] Cubas, Z. S. , J. R. Silva , and J. L. Catão‐Diaz . 2014. Tratado de animais selvagens. 2ª ed. Rocca.

[jez2922-bib-0016] Diniz, A. N. , G. T. Pessoa , L. da S. Moura , et al. 2017. “Computerized Electrocardiogram in Agoutis (*Dasyprocta prymnolopha* Wagler, 1831) Anesthetized With Ketamine and Midazolam.” Pesquisa Veterinária Brasileira 37: 150–155. 10.1590/S0100-736X2017000200009.

[jez2922-bib-0017] Diniz, A. N. , J. R. Silva Júnior , P. C. Guerra , et al. 2013. “Electrocardiogram Assessmenti in Non‐Anaesthetized Clinically Healthy Agouti (Dasyprocta Primnolopha, Wagler 1831).” Pesquisa Veterinária Brasileira 33: 8–14. 10.1590/S0100-736X2013001300002.

[jez2922-bib-0018] Fagundes, D. J. , and M. O. Taha . 2004. “Modelo animal de doença: critérios de escolha e espécies de animais de uso corrente.” Acta Cirurgica Brasileira 19: 59–65. 10.1590/S0102-86502004000100010.

[jez2922-bib-0019] Ferreira, L. M. , B. Hochman , and M. V. Barbosa . 2005. “[Experimental Models in Research].” Acta Cirurgica Brasileira 20, no. Suppl 2: 28–34. 10.1590/S0102-86502005000800008. 16283025

[jez2922-bib-0020] Filho, D. C. S. , and Â. V. F. Chaves . 2000. “O eletrocardiograma de alta resolução e suas aplicações clínicas.” Reblampa 13: 86–96.

[jez2922-bib-0021] Folino, A. F. , B. Bauce , G. Frigo , and A. Nava . 2006. “Long‐Term Follow‐Up of the Signal‐Averaged ECG in Arrhythmogenic Right Ventricular Cardiomyopathy: Correlation With Arrhythmic Events and Echocardiographic Findings.” EP Europace 8: 423–429. 10.1093/europace/eul035. 16690632

[jez2922-bib-0022] Frank, E. 1956. “An Accurate, Clinically Practical System for Spatial Vectorcardiography.” Circulation 13: 737–749. 13356432 10.1161/01.cir.13.5.737

[jez2922-bib-0023] Hasan, M. A. , D. Abbott , and M. Baumert . 2012. “Beat‐to‐Beat Vectorcardiographic Analysis of Ventricular Depolarization and Repolarization in Myocardial Infarction.” PLoS One 7: e49489. 23166683 10.1371/journal.pone.0049489 PMC3498118

[jez2922-bib-0024] Henkens, I. R. , K. T. B. Mouchaers , H. W. Vliegen , et al. 2007. “Early Changes in Rat Hearts With Developing Pulmonary Arterial Hypertension Can be Detected With Three‐Dimensional Electrocardiography.” American Journal of Physiology‐Heart and Circulatory Physiology 293: H1300–H1307. 17496210 10.1152/ajpheart.01359.2006

[jez2922-bib-0025] Ishikawa, K. , S. Handa , H. Nagoshi , et al. 1967. “A Study of the Normal Frank Vectorcardiogram.” The Keio Journal of Medicine 16: 13–21. 6065895 10.2302/kjm.16.13

[jez2922-bib-0026] Jaye, D. A. , Y.‐F. Xiao , and D. C. Sigg . 2010. “Basic Cardiac Electrophysiology: Excitable Membranes.” Cardiac Electrophysiology Methods and Models: 41–51. 10.1007/978-3-031-71067-4_2.

[jez2922-bib-0027] Júnior, A. M. C. , E. A. De Moura Fortes , D. J. A. De Menezes , L. De Oliveira Lopes , and M. A. M. De Carvalho . 2012. “Morphological and Morphometric Characterization of Agoutis’ Peripheral Blood Cells (*Dasyprocta prymnolopha*, Wagler, 1831) Raised in Captivity.” Microscopy Research and Technique 75: 374–378. 10.1002/jemt.21066. 21898666

[jez2922-bib-0028] Kück, K. , J. L. Isaksen , C. Graff , et al. 2018. “Spatial QRS‐T Angle Variants for Prediction of All‐Cause Mortality.” Journal of Electrocardiology 51: 768–775. 10.1016/j.jelectrocard.2018.05.011. 30177310

[jez2922-bib-0029] MacFarlane, P. W. 2012. “The Frontal Plane QRS‐T Angle.” Europace: European Pacing, Arrhythmias, and Cardiac Electrophysiology: Journal of the Working Groups on Cardiac Pacing, Arrhythmias, and Cardiac Cellular Electrophysiology of the European Society of Cardiology 14: 773–775. 22523378 10.1093/europace/eus057

[jez2922-bib-0030] Mady, C. , A. C. Barretto , P. J. Moffa , et al. 1985. “O vetocardiograma na forma indeterminada da doença de Chagas [The Vectorcardiogram in the Undetermined Form of Chagas’ Disease].” Arquivos Brasileiros de Cardiologia 44: 83–85. 4084089

[jez2922-bib-0031] Maheshwari, S. , A. Acharyya , M. Schiariti , and P. E. Puddu . 2016. “Frank Vectorcardiographic System From Standard 12 Lead ECG: An Effort to Enhance Cardiovascular Diagnosis.” Journal of Electrocardiology 49: 231–242. 10.1016/j.jelectrocard.2015.12.008. 26806119

[jez2922-bib-0032] Man, S. , A. M. Algra , C. A. Schreurs , et al. 2011. “Influence of the Vectorcardiogram Synthesis Matrix on the Power of the Electrocardiogram‐Derived Spatial QRS‐T Angle to Predict Arrhythmias in Patients With Ischemic Heart Disease and Systolic Left Ventricular Dysfunction.” Journal of Electrocardiology 44: 410–415. 10.1016/j.jelectrocard.2011.04.007. 21704219

[jez2922-bib-0033] Massone, F. 2011. Anestesiologia veterinária: Farmacologia e técnicas: Texto e atlas colorido. 1ª ed. Grupo Gen ‐ Guanabara Koogan.

[jez2922-bib-0034] De Moura, C. R. C. , C. R. A. Da Silva , F. L. Silva , and A. P. R. Costa . 2013. “Doppler Echocardiography and Electrocardiogram in Diagnosis of Dog in Pericardial Effusion: Case Report.” Acta Veterinaria Brasilica 7. http://periodicos.ufersa.edu.br/revistas/index.php/acta/article/view/3570/5256.

[jez2922-bib-0035] Narayanaswamy, S. 2002. “High Resolution Electrocardiography.” Indian Pacing and Electrophysiology Journal 2, no. 2: 50–56. 17006557 PMC1564053

[jez2922-bib-0036] Nava, A. 2000. “Signal‐Averaged Electrocardiogram in Patients With Arrhythmogenic Right Ventricular Cardiomyopathy and Ventricular Arrhythmias.” European Heart Journal 21: 58–65. 10.1053/euhj.1999.1733. 10610745

[jez2922-bib-0037] Oehler, A. , T. Feldman , C. A. Henrikson , and L. G. Tereshchenko . 2014. “QRS‐T Angle: A Review.” Annals of Noninvasive Electrocardiology 19: 534–542. 25201032 10.1111/anec.12206 PMC4237708

[jez2922-bib-0038] Pastore, C. , J. Pinho , C. Pinho , et al. 2016. “III Diretrizes da Sociedade Brasileira de Cardiologia sobre análise e emissão de laudos eletrocardiográficos.” Arquivos Brasileiros de Cardiologia 106: 1–23. 10.5935/abc.20160054 27096665

[jez2922-bib-0039] Scheffer, J. P. , A. Lacerda , D. A. Oliveira , et al. 2013. “Indução da miocardiopatia isquêmica em modelo experimental mini porco.” Brazilian Journal of Veterinary Research 35: 45–48. https://bjvm.org.br/BJVM/article/view/651.

[jez2922-bib-0040] Singh, S. , P. I. Johnson , R. E. Lee , et al. 1996. “Topography of Cardiac Ganglia in the Adult Human Heart.” The Journal of Thoracic and Cardiovascular Surgery 112: 943–953. 10.1016/S0022-5223(96)70094-6. 8873720

[jez2922-bib-0041] Sperelakis, N. , and L. Ramasamy . 2010. Propagation of Excitation in Cardiac Muscle Using Pspice Analysis for Simulated Action Potentials (500 p.).

[jez2922-bib-0042] Spier, A. W. , and K. M. Meurs . 2004. “Assessment of Heart Rate Variability in Boxers With Arrhythmogenic Right Ventricular Cardiomyopathy.” Journal of the American Veterinary Medical Association 224: 534–537. 10.2460/javma.2004.224.534. 14989545

[jez2922-bib-0043] Spinosa, H. , and F. Spinosa . 1991. “Sobre os efeitos farmacológicos da xilazina.” Biotemas 4.

[jez2922-bib-0044] Suzuki, Y. , A. C. Yeung , and F. Ikeno . 2008. “Importância dos estudos pré‐clínicos em animais de experimentação para a cardiologia intervencionista.” Arquivos Brasileiros de Cardiologia 91: 348–360. 10.1590/S0066-782X2008001700011. 19142381

[jez2922-bib-0045] Turrini, P. , A. Angelini , G. Thiene , et al. 1999. “Late Potentials and Ventricular Arrhythmias in Arrhythmogenic Right Ventricular Cardiomyopathy.” The American Journal of Cardiology 83: 1214–1219. 10.1016/S0002-9149(99)00062-4. 10215287

[jez2922-bib-0046] Wagner, A. E. , K. R. Mama , E. P. Steffey , L. F. Brevard , and P. W. Hellyer . 2002. “Behavioral Responses Following Eight Anesthetic Induction Protocols in Horses.” Veterinary Anaesthesia and Analgesia 29: 207–211. 10.1046/j.1467-2995.2002.00093.x. 28404364

[jez2922-bib-0047] Watkins‐Pitchford, J. M. 2010. “Butorphanol.” Essence of Analgesia and Analgesics 14: 154–156.

[jez2922-bib-0048] Wise, A. F. , T. M. Williams , M. B. G. Kiewiet , et al. 2014. “Human Mesenchymal Stem Cells Alter Macrophage Phenotype and Promote Regeneration via Homing to the Kidney Following Ischemia‐Reperfusion Injury.” American Journal of Physiology‐Renal Physiology 306: F1222–F1235. 10.1152/ajprenal.00675.2013. 24623144

[jez2922-bib-0049] Zacché, E. , T. C. A. de Assumpção , T. B. Corsini , and A. A. Camacho . 2017. “Time Domain Heart Rate Variability in Boxer Dogs With Arrhythmogenic Right Ventricular Cardiomyopathy.” Ciência Rural 47: 1–6. 10.1590/0103-8478cr20160740.

[jez2922-bib-0050] Zanos, P. , R. Moaddel , P. J. Morris , et al. 2018. “Ketamine and Ketamine Metabolite Pharmacology: Insights Into Therapeutic Mechanisms.” Pharmacological Reviews 70: 621–660. 10.1124/pr.117.015198. 29945898 PMC6020109

